# Assessing the kidney function parameters glomerular filtration rate and effective renal plasma flow with dynamic FDG-PET/MRI in healthy subjects

**DOI:** 10.1186/s13550-018-0389-1

**Published:** 2018-05-09

**Authors:** Barbara K. Geist, Pascal Baltzer, Barbara Fueger, Martina Hamboeck, Thomas Nakuz, Laszlo Papp, Sazan Rasul, Lalith Kumar Shiyam Sundar, Marcus Hacker, Anton Staudenherz

**Affiliations:** 1 Division of Nuclear Medicine, Department of Biomedical Imaging and Image-guided Therapy, Medical University of Vienna, Waehringer Guertel 18-20, 1090 Vienna, Austria; 2 Department of Biomedical Imaging and Image-guided Therapy, Division of General and Pediatric Radiology, Medical University of Vienna, Vienna, Austria; 3 Center for Medical Physics and Biomedical Engineering, Medical University of Vienna, Vienna, Austria

**Keywords:** FDG, PET/MRI, Glomerular filtration rate, Effective renal plasma flow

## Abstract

**Background:**

A method was developed to assess the kidney parameters glomerular filtration rate (GFR) and effective renal plasma flow (ERPF) from 2-deoxy-2-[^18^F]fluoro-d-glucose (FDG) concentration behavior in kidneys, measured with positron emission tomography (PET) scans.

Twenty-four healthy adult subjects prospectively underwent dynamic simultaneous PET/magnetic resonance imaging (MRI) examination. Time activity curves (TACs) were obtained from the dynamic PET series, with the guidance of MR information. Patlak analysis was performed to determine the GFR, and based on integrals, ERPF was calculated. Results were compared to intra-individually obtained reference values determined from venous blood samples.

**Results:**

Total kidney GFR and ERPF as estimated by dynamic PET/MRI were highly correlated to their reference values (*r* = 0.88/*p* < 0.0001 and *r* = 0.82/*p* < 0.0001, respectively) with no significant difference between their means.

**Conclusions:**

The study is a proof of concept that GFR and ERPF can be assessed with dynamic FDG PET/MRI scans in healthy kidneys. This has advantages for patients getting a routine scan, where additional examinations for kidney function estimation could be avoided. Further studies are required for transferring this PET/MRI method to PET/CT applications.

**Electronic supplementary material:**

The online version of this article (10.1186/s13550-018-0389-1) contains supplementary material, which is available to authorized users.

## Background

Glomerular filtration rate (GFR) and effective renal plasma flow (ERPF) are important clinical measures for general kidney functionality. They have a high clinical value for detection, treatment, and prevention of kidney disease. In nuclear medicine these, parameters can effectively be determined or assessed by examinations using different radio tracers [1, 2].

**Fig. 1:**
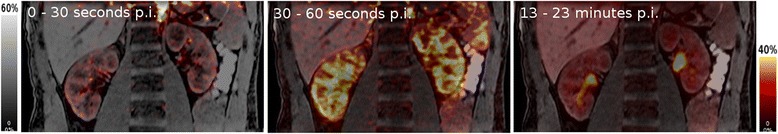
Images of FDG distribution in certain periods after injection (p.i.) obtained from a dynamic renal PET scan after image fusion with an MRI sequence

The most commonly used radio tracer for positron emission tomography (PET) examinations predominantly for oncological issues is the glucose analogue 2-deoxy-2-[^18^F]fluoro-d-glucose (FDG). FDG enters the kidney from blood vessels, is filtered in the glomeruli, is partially reabsorbed in the proximal tubule, and is finally excreted [3, 4]. Although it appears demanding to obtain information about renal function from a substance which is involved in that many different physiological processes, it would be of great advantage, if basic kidney function parameters, such as GFR and ERPF, could be extracted from the tracer’s behavior over time in a spatially resolved manner, allowing to even access the single kidney status. Because the according examination could happen within the accumulation time of FDG in the clinical routine, a determination of kidney functionality accompanying a routine dynamic FDG PET scan of the first 30 min after injection (p.i.) could save time and also applied radiation dose on patients (Fig. 1). This is of interest for patients, where kidney health status needs to be examined, e.g., in the case of patients getting a nephrotoxic chemotherapy.

Several approaches based on kinetic models [5, 6] have been proposed earlier to assess the renal clearance of FDG. Kinetic modeling approaches often require long scan times and are often followed by complex fitting routines to extract the kinetic parameters from the time activity curves (TACs). Fully integrated PET/MRI systems offer the possibility to perform simultaneous PET and magnetic resonance imaging (MRI) acquisitions in a single scan. The high-resolution MR volumes can be used to delineate different regions-of-interest, which in turn can be used to derive TACs from tissue to perform kinetic modeling. In this study, our aim was to find a simplistic clinically viable method to extract the kidney function parameters from routine PET/MRI scans.

## Methods

For the study, 25 adult and healthy subjects have been recruited; one female subject was excluded due to diabetes. All remaining 24 subjects fulfilled the requirements for the examinations (healthy condition, no metal in body, no claustrophobia, no pregnancy). Informed consent was obtained from all individual participants included in the study. They were examined twice between January and November 2016 at the General Hospital in Vienna. First is to obtain the reference values GFR_ref_ from drawn blood samples before and ERPF_ref_ from drawn blood samples after injection with ^99m^Tc-labeled mercaptoacetyltriglycine (MAG3). Secondly, 9 ± 5 days before or after that, a 30-min FDG PET/MRI scan was performed to obtain GFR_FDG_ and ERPF_FDG_. Basic subject data are summarized in Table 1.

**Table 1:** Subject demographics: basic subject data presented as mean value ± standard deviation, range from minimum to maximum value in parentheses

	24 subjects	Subject group 1	Subject group 2
Gender	18 male, 6 female	1 male, 2 female	6 male, 4 female
Age [years]	39 ± 14 (21–65)	42 ± 18 (31–63)	42 ± 17 (21–65)
Weight [kg]	85 ± 18 (50–120)	87 ± 18 (72–107)	78 ± 11 (61–161)
Height [cm]	180 ± 9 (161–200)	178 ± 4 (175–182)	179 ± 12 (161–200)
Creatinine [mg/dl]	0.9 ± 1.16 (0.54–1.21)	1.02 ± 0.28 (0.7–1.21)	0.84 ± 0.18 (0.54–1.07)

Subject group 1 was selected for reproducibility checks and subject group 2 to estimate aorta correction effects

### Reference examination protocol

First, a blood sample was drawn to determine hematocrit (Hct) and creatinine. All subjects underwent a routine dynamic renal scintigraphy according to the EANM standardized protocol [7] (for details, see below). In the course of this, another blood sample was drawn 41 ± 2 min after injection of around 80 MBq MAG3, which was used to determine MAG3 clearance. For this purpose, a standard of around 20 MBq in 1 ml was measured both in the dose calibrator for syringes and, after dilution by 1:100, in a gamma counter for blood sample measurement.

### PET/MRI examination protocol

**Fig. 2:**
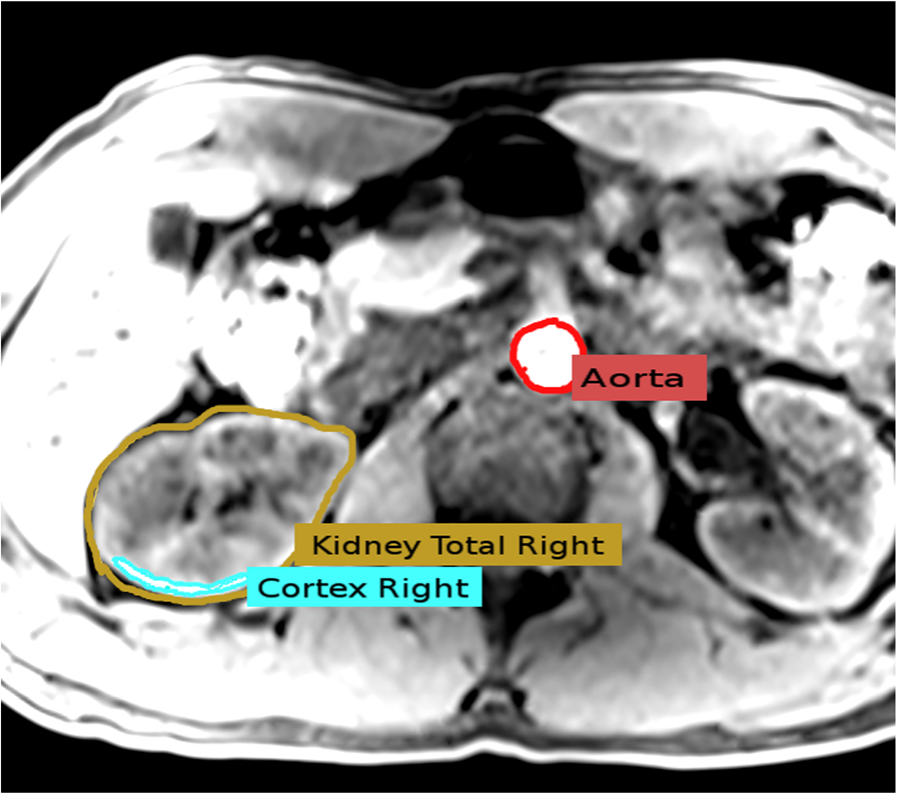
VOIs (volumes of interest) chosen in the T1 MRI sequence: total kidney region was selected in each layer, cortex region was chosen with threshold tool in several layers, and the aorta was chosen in the upper part of aorta descendens. Note that in this figure, aorta region is only seen for illustration purposes: aorta region taken for evaluation was chosen from super-incumbent segments. For the sake of visibility, the cortex region of interest (ROI) is located inside the total kidney ROI

Similar to renal scintigraphy, volunteers were hydrated with water (10 ml/kg body weight) for 20 min and asked to empty their bladder directly before injection of FDG (~ 3 MBq/kg body weight). The FDG is prepared in our institution on a routine basis to a well-known and established method [8] based on a GE FASTlab platform (General Electric Healthcare, USA). With a combined PET/MRI scanner (Siemens Biograph mMR, Siemens Healthcare Diagnostics GmbH, Germany), PET acquisition started immediately after tracer injection and continued for 30 min. The PET list-mode data was re-binned into a dynamic sequence: 60 × 5 s, 25 × 60s, and each PET frame was reconstructed (Siemens e7 tools) into a 172 × 172 × 127 matrix using the ordinary Poisson ordered subsect expectation maximization (OP-OSEM) 3D algorithm (3 iterations, 21 subsets, Gaussian filter). Scatter correction along with Dixon-based MR-attenuation correction was performed. The MR imaging protocol consisted of a T1 weighted MRI sequence (axial breath holding and fat suppression, VIBE SPAIR). A contrast-enhanced (Dotarem: 0.2 ml/kg body weight) TWIST dynamic MR sequence was performed on 10 subjects (group 2, see Table 1). To perform quantitative analysis, five volumes-of-interest (VOIs) were chosen with the Hermes Hybrid Viewer tool (Hermes Medical Solutions AB, Stockholm, Sweden): (1) *aorta descendens* (between diaphragm and arteria renalis), drawn by hand in several layers (2) left kidney, (3) right kidney, (4) left kidney cortex, and (5) right kidney cortex. VOIs (2–3) were carefully drawn by hand in each layer; VOIs (4–5) were delineated randomly in about 30% of all layers by threshold ROI selection tool in the outer part of the parenchyma. In Fig. 2, aorta, right cortex, and right total kidney ROIs are presented. VOIs were then copied to the PET images, from which the TACs, i.e., the FDG concentration in the VOIs over time, were exported in units of kilobecquerel per milliliter.

**Fig. 3:**
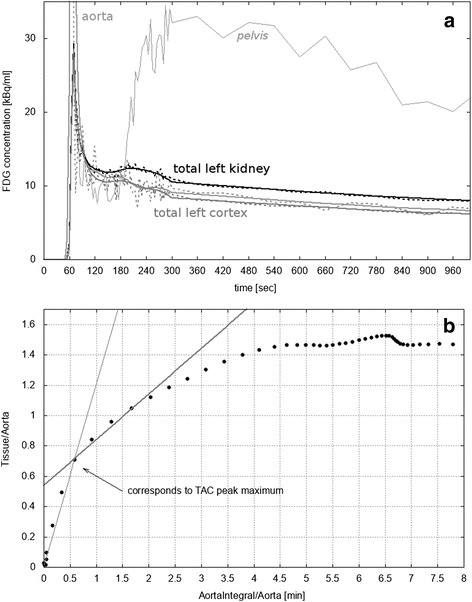
**a** Typical smoothed left cortex (gray), left total kidney (black), and aorta (light gray) time activity curves (TACs). For the sake of visibility, the aorta peak is outside of range (maximum 157.8 kBq/ml) and only the first 1000 s are plotted. Original curves are added as dotted lines. The TAC of the renal pelvis is indicated with thin gray lines. **b** Patlak plot of the left total kidney TAC. Two linear fits are shown: the first from the first point to that one which corresponds to the peak maximum (thin gray line) and the second covering the linear segment below 2 min starting at the point corresponding to the peak maximum (thick gray line, slope 0.3 min^−1^, offset 0.54). The latter was used to calculate the FDG glomerular filtration rate (GFR_FDG_)

FDG TAC analysis was performed using an in-house Java-based tool (programmed with openjdk version 1.8.0_162), for which the aorta input function (AIF) along with the TACs (Fig. 3) was used as inputs. With a machine learning approach (see Additional file 1 and [9]), it was found that TACs need to be smoothed with a filter for which a Bezier curve was calculated to overcome noise and fluctuations especially appearing in the initial part of the TACs. Since we observed a hump in the total kidney TACs between 3 and 5 min p.i, which was paralleled by an increase of concentration in the renal pelvis (see Fig. 3a), we assume that FDG remains in the kidney compartment during the first minutes (irreversible process within the minimal transit time) before it was forwarded into the pelvis or re-absorbed. Therefore, a graphical analysis was performed with a Patlak plot [10, 11].

### Determination of GFR and ERPF

The rapid decay of the peak might be affected by several processes, such as glomerular filtration, re-absorption, and forwarding to the renal pelvis. If many processes occur, a Patlak plot results in a complicated, curved shape [11], which can be clearly observed in Fig. 3b. To calculate GFR_FDG_, regression analysis was used to obtain the slope K of the Patlak plot (see Fig. 3b). To unambiguously identify the relevant linear part, a machine learning approach was used (see Additional file 1), showing that the linear part within the first 2 min, starting at the point which corresponds to the TAC peak maximum, was most suitable. Final GFR_FDG_ was then defined as the sum of the right and the left cortex or total kidney value *V*:

GFR_FDG_[ml/min] = *K*_right_[min^−1^]*V*_right_[ml] + *K*_left_[min^−1^]*V*_left_[ml]

As reference value, GFR_ref_ was estimated from creatinine values with the CKD-EPI formula [12].

Initial FDG blood flush is represented by the integral of the TAC peak, while the TAC peak maximum *P*_max_ gives the largest by the kidney physically graspable quantity of fluid. The ratio between these two quantities consequently results in a time value representing the capability of the kidney to forward the maximum quantity of fluid and was therefore taken as a measure for the ERPF, i.e., ERPF_FDG_. This value was then multiplied with the corresponding kidney volume *V*. Final cortex and total kidney ERPF_FDG_ were taken as sum of the corresponding left and right single kidney values.

$$ {\mathrm{ERPF}}_{\mathrm{FDG}}\left[\mathrm{ml}/\min \right]=\frac{P_{\mathrm{max}}^{\mathrm{right}}\left[{\mathrm{ml}}^{-1}\right]}{\int_{\mathrm{Peak}}{\mathrm{TAC}}_{\mathrm{right}}\left[{\mathrm{ml}}^{-1}\min \right]}{V}_{\mathrm{right}}\left[\mathrm{ml}\right]+\frac{P_{\mathrm{max}}^{\mathrm{left}}\left[{\mathrm{ml}}^{-1}\right]}{\int_{\mathrm{Peak}}{\mathrm{TAC}}_{\mathrm{left}}\left[{\mathrm{ml}}^{-1}\min \right]}{V}_{\mathrm{left}}\left[\mathrm{ml}\right] $$

The integral over the peak was calculated from peak rise to 1 min after peak rise, which was found with the machine learning approach.

MAG3 clearance and, from this, ERPF_ref_ were determined from one blood sample, drawn ~ 40 min p.i. according to [1, 13].

### Error estimation

For reference values, GFR_ref_ error was set to 12% according to [14]. ERPF_ref_ error was estimated with 10%, because the blood sample measurement protocol was identical to [15], showing that errors, usually not higher than 10%, mainly arise from the measurement procedure.

To estimate errors of GFR_FDG_ and ERPF_FDG_, the reproducibility of the VOI choice for FDG TAC analysis was assessed with data from subject group 1 (Table 1), having high differences between their reference values, age, and gender. For each subject, the above described procedure for calculating GFR_FDG_ and ERPF_FDG_ was repeated using three different VOIs for each aorta, cortex, and total kidney, leading to seven different combinations of cortex/total kidney TACs and AIF. Different layers, VOI sizes, and regions have been chosen to alter the aorta and cortex VOIs. Note that the aorta VOIs were still located between diaphragm and arteria renalis. Either total kidney VOIs were delineated more inaccurately or every second layer was used for manual delineation, and a Hermes Hybrid Viewer tool was used to automatically fill up the missing layers. For each subject, the deviations from the mean GFR_FDG_ and ERPF_FDG_ were then calculated from all different VOIs and averaged over all three subjects.

In the case of GFR_FDG_, total error was taken as square root of the squared sum of all contributing errors, which was the standard error from just described reproducibility checks as well as the standard error from the linear fit of the Patlak plot.

### Estimation of AIF correction effects

**Fig. 4:**
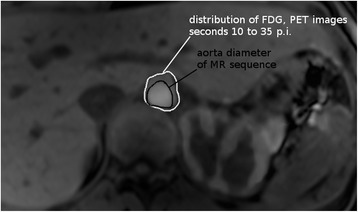
Fused image of MRI T1 sequence and PET. From the latter, the sum of the second to seventh frame (i.e., seconds 10 to 35) p.i. is shown. Due to partial volume effects, the FDG distribution in the PET images (white contour) clearly exceeds the aorta diameter observed with MRI image (black contour). Also, the initial accumulation of FDG in the cortex is visible

After image fusion, it was observable that the FDG distribution in the PET images was blurred, i.e., it exceeded the border of the aorta region in the MRI scans, in particular during the first frames (see Fig. 4), mainly due to motion and partial volume effects. Due to these effects, FDG concentration was falsely spread over a larger volume, leading to an underestimation of concentration. The effect was studied on the basis of a method described by Khalighi et al. [16, 17] with the dataset of the subjects from subject group 2, who additionally had contrast-enhanced MRI examination. The active contour algorithm from ITK snap (version 3.6.0) was used to extract the aorta volume V_A_ from the contrast-enhanced MR data. Spill-out region was defined by summing up the early PET frames and segmenting the aorta from the summed PET images. Using Matlab R2016a (MathWorks, USA), a background sampling region was defined by radially dilating the spill-out mask by three voxels (12 mm); the activity *C*_B_ and the volume *V*_B_ in this region were measured. Similarly, the volume of the aorta, *V*_A_, was also determined. The total area comprises of the spill-out and background region with activity *C*_T_ and volume *V*_T_. The activity balance in these three volumes can be then modeled by *C*_T_*V*_T_ = *C*_A_*V*_A_ + *C*_B_*V*_B_. Finally, this equation was solved for *C*_A_, the FDG tracer concentration in the aorta (AIF), and was repeated for all frames. GFR_FDG_ from cortex and total kidney TACs of each subject was then re-calculated with the corrected AIF. The deviation of the GFR_FDG_ obtained from the corrected AIF to the uncorrected AIF was calculated, averaged over all subjects and expressed in percent.

### Statistical evaluation

Statistical analysis was performed with Gnumeric (open source software, version 1.12.20) and LibreOffice Calculator (open source software, version 4.3.7.2). First, reference values, values from FDG TACs, and basic subject data were tested for normal distribution with Kolmogorov-Smirnov test. Correlations have been calculated with Pearson product-moment correlation coefficient *r* from which *p* value was derived. The significance of the differences between reference and FDG value was assessed by a paired Student’s *t* test (*p* < 0.05 was considered as a statistically significant difference).

## Results

Subject demographics are summarized in Table 1. Total evaluation time per subject was 80 min (blood sample measurement and ERPF_ref_/GFR_ref_ calculation: 60 min, FDG TAC extraction and analysis: 20 min). One male subject had horseshoe kidneys, and in another male subject, Tarlov cysts were found in the lower back, both without any health effects. Kolmogorov-Smirnov tests delivered a possible normal distribution for all evaluated parameters, except age, gender, and total kidney GFR_FDG_.

### GFR and ERPF

**Fig. 5:**
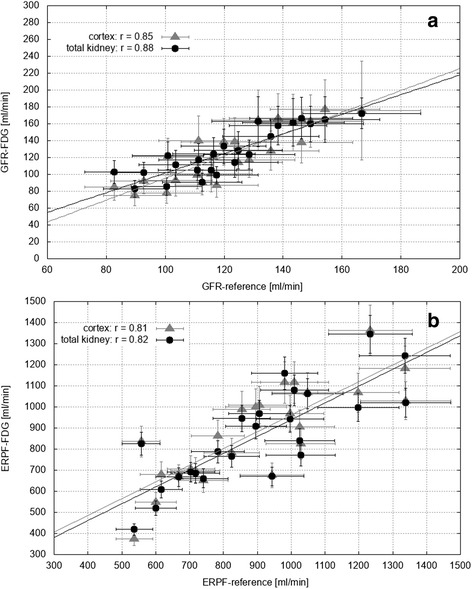
Reference values versus values obtained from total kidney (black) and cortex (gray) FDG TACs (time activity curves), for **a** GFR (glomerular filtration rate) and **b** ERPF (effective renal plasma flow). Lines of best fit are also shown. Error bars represent the calculated errors of each data point

As shown in Fig. 5, cortex GFR_FDG_ showed an excellent correlation with GFR_ref_ (*r* = 0.85; *p* < 0.0001) and total kidney GFR_FDG_ showed an excellent correlation with GFR_ref_ (*r* = 0.88; *p* < 0.0001). ERPF_FDG_ correlated with cortex ERPF_ref_ by *r* = 0.81 (*p* < 0.0001) and with total kidney by *r* = 0.82 (*p* < 0.0001) (see Fig. 5).

The linear part of the Patlak plot below 2 min was used for the linear regression to obtain GFR_FDG_. It should be noted that using more fit points of the Patlak plot up to 4 min did not change the correlation significantly (*r* > 0.84), but the differences between the means of GFR_FDG_ and GFR_ref_ were higher (+ 65 ml/min in average).

**Fig. 6:**
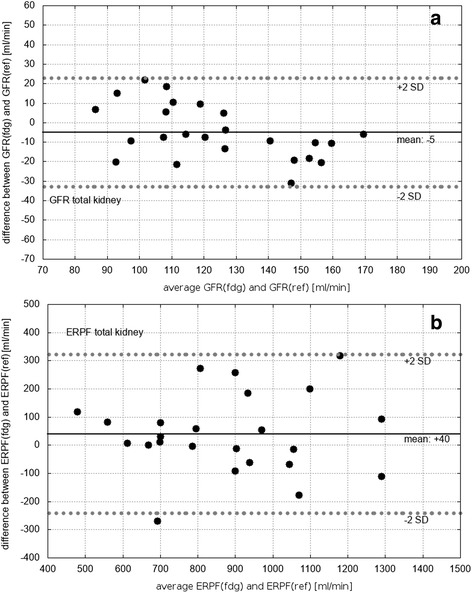
Bland-Altman-plots of **a** glomerular filtration rate (GFR) and **b** effective renal plasma flow (ERPF), only shown for total kidney results. Black lines represent the mean deviation and dashed lines the mean value ± 2 standard deviations (SD)

All results as well as differences from the Bland-Altman analysis are summarized in Table 2 and Figs. 5 and 6. Paired Student’s *t* test showed no significant difference between FDG and reference values, neither for cortex and total kidney values nor for GFR and ERPF.

**Table 2:** Main results: mean values ± standard deviation (SD) as well as total errors for glomerular filtration rate (GFR) and effective renal plasma flow (ERPF)

	Mean ± SD (min–max)	Total error (%)	*r*	BA difference
GFR reference value [ml/min]	122 ± 21 (83–167)	12		
GFR FDG value (total) [ml/min]	127 ± 28 (83–172)	(11–18)	0.88	− 5 ± 14
GFR FDG value (cortex) [ml/min]	123 ± 33 (75–177)	(16–33)	0.85	− 2 ± 18
ERPF reference value [ml/min]	898 ± 234 (537–1338)	10		
ERPF FDG value (total) [ml/min]	858 ± 229 (419–1345)	7	0.82	+ 40 ± 141
ERPF FDG value (cortex) [ml/min]	880 ± 229 (375–1364)	9	0.81	+ 18 ± 143

Ranges from minimum (min) to maximum (max) value are indicated in parentheses. Column 3 presents the Pearson product-moment correlation coefficient *r* between FDG and reference values and column 4 the differences according to the Bland-Altman (BA) analysis

Total errors are also summarized in Table 2 and presented as error bars in Fig. 5. Note that total error of GFR_FDG_, in contrary to ERPF_FDG_, arises not only from reproducibility checks but also from Patlak fit errors, leading to an individual error for each value which is indicated as range in Table 2. Reproducibility checks showed a variation of 7% in case of total kidney and 11% in case of cortex GFR_FDG_ and a variation of 7% in case of total kidney and 9% in case of cortex ERPF_FDG_. Furthermore, no significant difference was found within subject group 1. Accuracy of kidney volume determination was found to be 6%.

### Estimation of aorta correction effects

The effect of the observed blurring of the FDG distribution in the aorta region was estimated with a corrected AIF. GFR_FDG_ varied in the case of cortex by (6 ± 17) % in case of total kidney by (− 1 ± 7) %. Note that ERPF_FDG_ is not affected by this correction because its calculation does not depend on the AIF.

## Discussion

The main result of our study was that GFR and ERPF can be accurately calculated from renal and aortic FDG TACs obtained by dynamic PET/MRI scans.

FDG excretion from TACs of kidneys, aorta, and bladder was previously studied with a detailed kinetic model in mice treated with metformin [6], where an excellent correlation (*r* = 0.95) was obtained between the rate coefficient associated with GFR and urinary clearance. Although the computation seems to be robust with respect to initialization, this model needs a sophisticated algorithm and is based on seven rate coefficients (to be fitted) describing many renal processes, which might overshoot our challenges. Furthermore, a PET field of view covering aorta descendens, kidneys, and bladder in humans is not practical to realize, and the kinetic model was optimized for mice under medical treatment. Another approach considering bladder TACs and integrals over the AIF was also performed in mice [18]. The obtained FDG clearance correlated well with MAG3 tubular extraction rate (*r* = 0.73) and with creatinine clearance (*r* = 0.78) from blood samples. However, despite of a challenging broad field of view, a model for mice under anesthesia cannot be directly transferred to humans. For human studies, a kinetic model using a delay constant and less rate coefficients was developed by Qiao et al. [5]. Plasma clearance was estimated from FDG excretion by multiplying the kidney volume with the corresponding rate coefficient after fitting the model to total kidney TACs. Not unusual for kinetic modeling, long scan times of 60 min were used. With datasets of 10 humans, a deviation of 10% was obtained compared to normal GFR value (125 ml/min), which was used as reference value.

Our aim was to assess kidney function without complex models or fitting algorithms. In a simplified understanding of the renal FDG processes, the TAC peak represents the initial blood flush and therefore ERPF, while its decay reflects subsequent processes, e.g., re-absorption, forwarding, and also glomerular filtration (and therefore GFR). We observed a hump between 3 and 5 min p.i. in all total kidney TACs, which was accompanied by an increase in FDG concentration in the renal pelvis. We therefore concluded that FDG is trapped in the kidney during the first minutes, allowing to apply a Patlak plot analysis to study initial processes, such as glomerular filtration. Certainly, the usage of the first 2 min of the Patlak plot lead to a low set of data points to be fitted and therefore relatively high fit errors. However, both GFR_FDG_ and ERPF_FDG_ derived from this approach showed an excellent correlation with the reference methods and small difference between their means. Furthermore, only small differences between cortex and total kidney results were noted. In particular, errors arising from reproducibility checks were higher in the case of cortex values, indicating that cortex TACs are in general less reliable (probably due to partial volume and motions effects). Thus, total kidney TACs were sufficient to assess GFR and ERPF. Moreover, even inaccurately drawn VOIs resulted in a deviation of 7% in total kidney GFR_FDG_ and ERPF_FDG_, a detailed and time-consuming drawing of total kidney VOIs thus appears not necessary and assisting tools such as interpolation of ROIs can be used. Blurring effects of the FDG distribution around the aorta showed a negligible effect of − 1% in the case of total kidney after an appropriate correction; therefore, it can be disregarded for the GFR_FDG_ calculation.

The described Patlak analysis is only valid for our reconstruction conditions and chosen aorta VOI, because the Patlak plot shape—and therefore the calculated GFR_FDG_—strongly depends on the time binning of the TACs and (due to partial volume effects) on the position of the aorta VOI. Note that the *aorta descendens* was chosen to minimize (a) partial volume effects because of the wide aorta diameter, (b) motion effects because of mainly axial movement in this aortic area, and (c) disturbances from heart activity because of its position located beneath the heart.

The length of linear fit in the Patlak analysis might be adopted for different conditions. Consequently, the presented methods for GFR and ERPF calculation might be transferred to other PET modalities. Furthermore, since only the peak is used for all calculations, PET acquisition time can be reduced to below 10 min.

### Limitations

There are several limitations of the present study, which have to be mentioned.

Firstly, the reference value GFR_ref_ was estimated from creatinine level in the blood to keep the subject comfort in mind [19]. Clearance of MAG3 is usually connected to the so-called tubular extraction rate, but it can be used as a reliable estimator of ERPF after a conversion [13, 20].

Secondly, FDG physiology is complex, a simple approach as the presented one therefore has obvious shortcomings, especially if one of the initial processes forming the peak shape is affected, e.g., in the case of chronic kidney diseases or in the case of diabetes where renal glucose re-absorption is changed [21]. The current study was based on healthy subjects; the efficiency of the method needs to be evaluated in the context of patients with pathology.

Thirdly, the method according to [16] used to correct partial volume effects in the aorta might only be applicable to PET/MRI scans, for which this method was proven to be reliable [16, 17] with even thinner cervical arteries used for the AIF.

Regarding a possible transfer of our method to different PET modalities, we focused in the present study on an evaluation of PET data in comparison to reference values. Due to this reason, the MRI part of our PET/MRI system was only used to separate renal cortex from other renal parts, even if several methods exist to obtain reliable values for kidney function parameters such as renal blood flow [22] or in particular GFR [22–25] with different sequences, with contrast agents, based on Patlak plots or kinetic models.

## Conclusions

In summary, we present a proof of concept to assess total and even single kidney GFR and ERPF from a single dynamic FDG PET. For clinical usage, total kidney VOI and a PET acquisition time of a few minutes are sufficient. This allows a simultaneous estimation of relevant kidney function parameters within a routine PET scan. Further studies are required for transferring this PET/MRI method to PET/CT applications and to check the findings in the case of insufficient kidney function.

## Additional file

Additional file 1: FDG TAC analysis. (DOCX 11 kb)

## Abbreviations

AIF: Aorta input function
BA: Bland-Altman
ERPF: Effective renal plasma flow
FDG: 2-Deoxy-2-[^18^F]fluoro-d-glucose
GFR: Glomerular filtration rate
MAG3: ^9m^Tc-labeled mercaptoacetyltriglycine
ML: Machine learning
PET/MRI: Positron emission tomography/magnetic resonance imaging
TAC: Time activity curve
