# Supplementary material for: Assessing the kidney function parameters glomerular filtration rate and effective renal plasma flow with dynamic FDG-PET/MRI in healthy subjects
Source: EJNMMI Res. 2018 May 9;8:37. doi: 10.1186/s13550-018-0389-1 (PMC5943199; doi:10.1186/s13550-018-0389-1)
Supplement: Supplementary file 1 — FDG TAC analysis. (DOCX 11 kb) [file 13550_2018_389_MOESM1_ESM.docx]

**SUPPLEMENT**

FDG TAC analysis

FDG TAC analysis was performed with a Java-based, self-written program*.* The software was fed with TACs of left and right cortex and total kidney, as well as of the aorta (i.e. the image derived AIF), which were exported from *Hermes Hybrid Viewer.*

In order to calculate GFR and ERPF from FDG TACs, the latter had to be adapted and constraints for an automatic procedure had to be found. Therefore, different combinations of variables (in detail described below) in the source code of the software had to be permuted and the in each case obtained GFR and ERPF values were compared to the according reference values. For this, a covariance matrix of FDG and reference kidney parameters was calculated, allowing to search for a maximum number of high correlations (r > 0.7). This tedious procedure was done with a machine learning (ML) approach, built on the principles of Genetic Algorithms (GA) [9].

The software had five unknown parameters that affected the TAC preparation procedure: the type of the filter, the starting point of the filter calculation, two parameters for the Patlak plot (see below), as well as the length of the peak integral. A value range with finite parameter choices was defined for each of the unknown parameters in order to regularize the optimization procedure. Each parameter was characterized by its non-negative index inside its respective range, thus, the GA approach was operated with non-negative index sets. In the initialization stage the GA optimizer built up a random population of parameters index sets. Whenever a new parameter index was created by the GA, the software was executed with the respective parameters which consecutively processes the TACs of the subjects and calculated GFR and ERPF. Note that this procedure was performed before the very end of the study, therefore a sufficient amount of 22 subject data were used. The fitness value of the parameter index set was provided by the sum of high correlations (r > 0.7) in the covariance matrix. The GA optimizer was built on an iterative approach in which a new population of parameter index sets had to be created from the current population. This process was performed by the fitness-driven tournament selection as well as crossover of two parameter index sets to create a new “offspring” index set. Random index values of the resulted offspring were further mutated. The mutation step was regularized in order to keep the parameter indices inside the preferred ranges. The resulted offspring was placed in the new population with its evaluated fitness values. This procedure was repeated until the new population was filled. The described GA iteration scheme was performed in 100 cycles. This procedure also allowed to search for hidden correlations between basic patient data and kidney parameters extracted from FDG TACs (which could not be found). After 100 GA cycles, an optimum set of variables corresponding to the fittest parameter was identified allowing to automatically calculate GFR_FDG_ and ERPF_FDG_.

Smoothing: In order to overcome noise and fluctuations especially appearing in the initial part of the TACs, they had to be filtered. Optimum results have been obtained when calculating the Bezier curve starting at the peak maximum of the corresponding TAC (dashed and dotted lines in fig. 3a). This was found with ML after varying between no filter, Bezier filter and Savitzky-Golay filter. Also the starting point of the filter procedure has been varied between peak maximum and the first point after the peak decay passing into the plateau region of the TAC.

Integrals: For the ERPF calculation, the integral over the TAC peak was used, covering the first 60 seconds after peak rise. This was found with ML after varying the integral time between 30 seconds and 180 seconds.

Patlak plot: This graphical analysis technique is based on the equation [10,11]:

$\frac{T\left( t \right)}{A\left( t \right)}=K\frac{\int_{0}^{t} A\left( \tau\right)d\tau}{A\left( t \right)}+V_{0}$

with T(t) as the amount of tracer in the tissue, A(t) as the concentration of tracer in the blood and V_0_ as the intercept partially containing the effective plasma volume [10]. The clearance K can be assessed by linear regression from a graph, where the T(t)/A(t) is plotted against $\frac{\int_{0}^{t} A\left( \tau\right)d\tau}{A\left( t \right)}$.

Due the complex shape of the Patlak plot (see fig. 3b and [11]), the ML approach was used to identify the relevant linear part: The starting point of the linear fit, as well as the number of data points to be fitted were varied. With this automatic routine, best correlation and lowest difference between the mean values of GFR_FDG_ and GFR_ref_ were found when the fit started at that data point which corresponded to the TAC peak maximum and when the length of the slope did not exceed 2 minutes on the abscissa. Therefore, this linear part after the starting point was used for the regression analysis.
